# Enhancing Effect of Coupling Agent Sizing on the Mechanical Properties of Carbon Fiber Reinforced Acrylonitrile-Butadiene-Styrene Composites

**DOI:** 10.3390/ma19061147

**Published:** 2026-03-15

**Authors:** Youqiang Yao, Xiaoqing Fang, Zhonglue Hu, Weiping Dong, Bin Wang, Sisi Wang, Xiping Li

**Affiliations:** College of Engineering, Zhejiang Normal University, Jinhua 321004, China; yaoyouqiang@zjnu.edu.cn (Y.Y.);

**Keywords:** carbon fiber reinforced, ABS, interface bonding, surface modification

## Abstract

**Highlights:**

**What are the main findings?**

- KH792 builds hybrid Si–O–C/Si–O–Si interface on CF, with –NH_2_ bridging to ABS.
- Modified CF has <20° contact angle, higher surface energy; T_onset +17 °C, T_max +10 °C.
- CF/ABS tensile strength: 58.41 MPa (injection) and 49.37 MPa (3D-printed).

**What are the implications of the main findings?**

- KH792 covalent anchoring enhances fiber-matrix interfacial bonding effectively.
- Higher thermal stability broadens application potential in heat-resistant components.
- High-performance CF/ABS achieved via both molding and additive manufacturing.

**Abstract:**

This study investigates the influence of surface-modified carbon fibers (CFs) on the structural and mechanical properties of acrylonitrile-butadiene-styrene (ABS)-based composites. A comprehensive approach employing Fourier Transform Infrared Spectroscopy (FTIR), contact angle measurement, and thermogravimetric analysis (TGA) characterized the CF surface chemistry, wettability, and thermal stability. Specimens were prepared via injection molding and 3D printing processes, enabling systematic evaluation of tensile, flexural, and impact properties. Combined with Scanning Electron Microscopy observations of composite fracture surfaces, the study elucidates how modification treatments influence fiber–matrix interface bonding and mechanical enhancement mechanisms. The results indicate that after resizing treatment with silane coupling agents, the surface activity of CF and its interfacial compatibility with ABS were significantly improved, leading to a marked enhancement in the composite material’s overall performance. At a CF content of 9.62 wt%, the ABS-S-CF2 system exhibited optimal mechanical properties: The tensile strength and flexural strength of the injection-molded specimens reached 58.41 MPa and 81.51 MPa, respectively, representing increases of approximately 41.6% and 29.1% compared to neat ABS. The tensile strength and flexural strength of the printed specimens also reached 49.37 MPa and 80.19 MPa, respectively. Microstructural analysis indicates that the sizing treatment improves the interfacial bonding between CF and neat ABS.

## 1. Introduction

Carbon Fiber Reinforced Thermoplastic Composites (CFRTPCs) combine the high modulus characteristics of carbon fibers with the toughness of a thermoplastic resin matrix. Consequently, they exhibit advantages such as high specific strength, high specific modulus, recyclability, and high durability [1]. Their application in fields like aerospace, new energy vehicles, and medical devices is steadily increasing [2,3]. Acrylonitrile-butadiene-styrene (ABS) is an outstanding ternary thermoplastic polymer offering excellent comprehensive properties derived from its three constituent monomers. The acrylonitrile (AN) component provides chemical corrosion resistance through its strongly polar cyano group. The butadiene (BD) component forms a rubber phase that acts as a stress concentrator, effectively absorbing impact energy. The styrene (St) component imparts good processing stability to the material. With the rapid development of industrial technology towards high performance and lightweight design, research has been conducted to optimize the fundamental properties of traditional ABS materials through the addition of phases. Carbon fiber possesses advantages such as high modulus, high specific strength, high-temperature resistance, and corrosion resistance, making it commonly used as a reinforcing material in composite structures.

Therefore, combining carbon fibers with ABS for composite reinforcement serves to broaden their application in equipment manufacturing by better meeting the demanding material performance requirements.

Due to the smooth surface and high chemical inertness of carbon fibers, their interfacial bonding with ABS resin polymer, which is non-polar or weakly polar [2], is poor. This hinders stress transfer efficiency and readily initiates fiber debonding at the interface, ultimately reducing the overall performance of the composite material [3,4]. The issue of interfacial compatibility is a key factor constraining the performance enhancement of CF/ABS composites. Research on improving CF-containing composites has been documented in the literature, employing methods such as plasma treatment [5,6,7], surface functional group grafting [8,9,10,11], surface sizing treatments [12,13,14], and surface oxidation [15,16]. For instance, Alinda et al. [17] significantly enhanced the tensile strength and impact resistance of carbon fiber-reinforced ABS composites by introducing fullerene (C60) nanoparticles into short carbon fibers, blending them with ABS, and then injection molding the composite. Li et al. [12] incorporated hydroxyl-functionalized boron nitride (BNO)-grafted carbon nanotubes (BNO-CNT) into a polyethyleneimine (PEI) sizing agent. This approach effectively enhanced the surface roughness and surface energy of carbon fiber (CF), while providing abundant oxygen-containing functional groups. These modifications promoted interfacial mechanical interlocking and bonding within the CF/PEEK composite. The resulting reinforced composite particles demonstrated significant improvements in flexural strength, flexural modulus, and interfacial shear strength (ILSS) by 77.71%, 59.39%, and 68.93%, respectively.

Therefore, sizing agents are recognized as an effective method for improving the interfacial properties between CFs and polymers. Sizing agents can form chemical bonds or physical adsorption layers on the CF surface, enhancing the wettability and interfacial adhesion between the fiber and the matrix. However, commercially available carbon fiber products typically feature functional sizing treatments primarily designed for thermoset composites [18] (such as epoxy resin matrices). As demonstrated by Peter et al. [19], epoxy functional groups in thermoset resins can form chemical bonds with the sizing agent through cross-linking reactions, resulting in strong and tough interfacial bonding. AHMED et al. [20] investigated the effect of polyurethane (PU) sizing agent on the mechanical properties of CF/ABS composites. The PU-sized CF composites exhibited higher tensile strength and modulus. To achieve optimal interfacial performance, the design of the sizing agent must match the chemical characteristics of the target polymer matrix. Consequently, for the preparation of CF/ABS composites, a process is employed where the fiber surface is first desized. A new sizing layer is then introduced to replace thermoset-compatible sizings. This newly sized fiber is subsequently melt-compounded with acrylonitrile-butadiene-styrene (ABS), ultimately enhancing the composite’s interfacial bonding and mechanical properties.

This study proposes a combined surface modification strategy of acetone heating activation and KH-792 resizing, aiming to achieve precise regulation of carbon fiber surface properties. Meanwhile, this study systematically explores the adaptability of modified CF/ABS composites in different molding technologies through injection molding and fused deposition modeling (FDM) processes for the first time. Furthermore, this study comprehensively employs multiple characterization techniques, including Fourier Transform Infrared Spectroscopy (FT-IR), contact angle measurement, thermogravimetric analysis (TGA), and Scanning Electron Microscopy (SEM), to reveal the intrinsic mechanism of interfacial reinforcement from the perspective of chemical bonds and microstructure. The results of this study can provide a theoretical basis for the design and application of high-performance CF/ABS composites.

## 2. Materials and Methods

### 2.1. Materials

Acrylonitrile butadiene styrene (ABS HI100) was obtained from LG Chem in Seoul, South Korea, with a density of approximately 1.02 g/cm^3^ and a melt flow index (MFI) of 11 g/10 min (220 °C/10 kg). The carbon fiber bundle was purchased from Zhongfu Shenying Carbon Fiber Co., Ltd., Lianyungang, China. with an average diameter of 7 μm. The diamino-functional silane KH792 (assay > 98%) was used, purchased from Kangjin New Materials Technology Co., Ltd., Dongguan, China.

### 2.2. Surface Modification of Carbon Fibers

**Figure 1:**
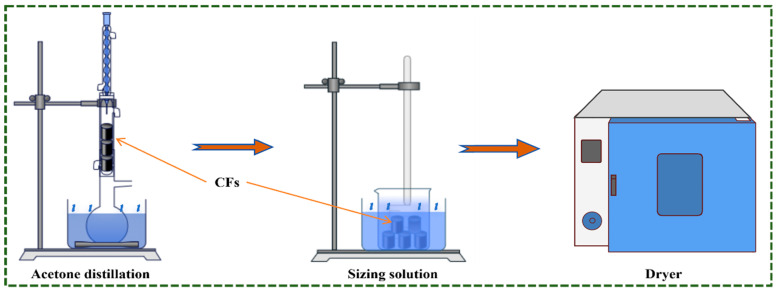
Schematic showing sizing treatment on CFs surfaces.

The surface treatment of carbon fibers employs a stepwise solution impregnation method, with the specific process illustrated in Figure 1. Commercial carbon fibers are first refluxed in acetone at 80 °C for 24 h to remove the original sizing agent and impurities from their surfaces. Subsequently, they are rinsed three times with deionized water to obtain desized carbon fibers (denoted as T-CFs). Place T-CFs in a solution composed of 10 phr hydrogen peroxide and 2 phr sodium hydroxide. Subject the mixture to oxidation treatment at room temperature for 12 h. To further promote hydrolysis, add dilute hydrochloric acid dropwise to adjust the solution pH to approximately 4, thereby completing the oxidation activation process. Finally, prepare a mixed solvent by combining anhydrous ethanol and deionized water in an 80:20 volume ratio. Add 5 phr of N-β-aminoethyl-γ-aminopropyltrimethoxysilane (KH-792) and stir thoroughly. The oxidized CFs were immersed in this solution and reacted at 60 °C in a water bath for 4 h. After removal, they were dried in an 80 °C oven for 5 h to obtain resizing carbon fibers, denoted as S-CFs. Untreated raw carbon fibers are denoted as C-CFs and serve as the control.

### 2.3. Preparation of CF/ABS Composites

**Figure 2:**
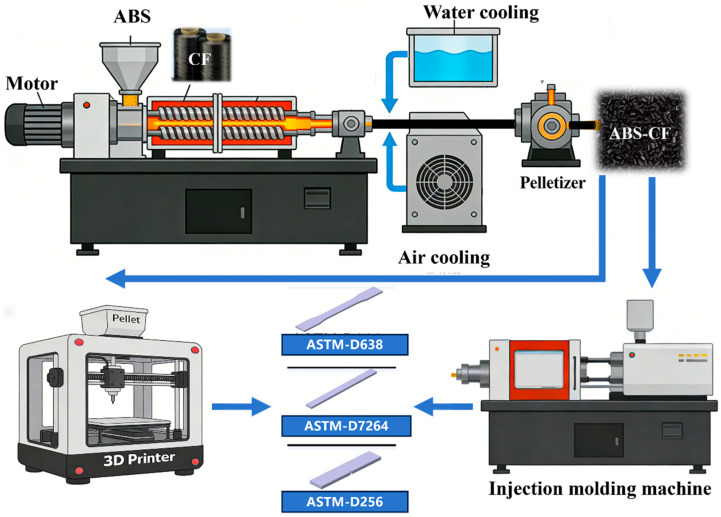
Process flowchart for preparing ABS composite materials and schematic diagram for standard specimens.

ABS was dried in an 80 °C oven for 6 h to remove moisture. Subsequently, ABS was melt-blended with C-CF, T-CF, and S-CF materials in specific ratios through an extruder to produce composite materials. As shown in Figure 2, ABS was melt-blended using a twin-screw extruder (SHJ-20, Nanjing Shengchi, Nanjing, China). The extruder zone temperatures were set to 200, 220, 220, 225, 225, 230, and 220 °C. Fiber content was controlled by varying the number of carbon fiber strands. The melt underwent water cooling and air cooling before being pelletized using a pelletizer to produce different ABS composites. During testing, C-CF/ABS, T-CF/ABS, and S-CF/ABS samples were simultaneously prepared.

The test specimens were injection-molded using an injection molding machine (Guangdong Yizumi Precision Machinery Co., Ltd., Foshan, China), with parameters set at 245, 245, 245, 245, 245, 245, and 245 °C to complete the injection molding of the test plates.

The test sample was successfully 3D-printed using a granular 3D printer (manufactured by Zhejiang Chaoling Technology Co., Ltd., Jinhua, China) with a nozzle diameter of 1.5 mm. Printing parameters were set as follows: nozzle temperature 240 °C, layer height 0.5 mm, retraction angle 0°, and infill density 100%.

### 2.4. Characterizations

In this experiment, injection molding and 3D printing experiments were conducted separately.

FT-IR spectroscopy was performed using a Fourier Transform Infrared Spectrometer (Nicolet iS5, Thermo Fisher, Waltham, MA, USA) to obtain FT-IR spectra of the samples for determining the surface chemical properties of CF. Analysis was conducted in the wavenumber range from 600 to 4000 cm^−1^ with a resolution of 4 cm^−1^.

The average contact angle of deionized water on CF bundles was measured using a contact angle measuring instrument (JC2000C1, Shanghai Aifisi Precision Instrument Co., Ltd., Shanghai, China). Different experimental CF samples were fixed onto microscope slides. Using a syringe to control the dispensing volume at 3 μL, the prepared liquid sample was transferred and slowly added to the surface of the experimental sample under test. The droplet formed a spherical shape due to its own surface tension, enabling contact angle measurement and calculation. Each sample was tested three times, with the average of three readings taken.

Thermogravimetric analysis (TGA) was performed using a thermogravimetric analyzer (PE TGA4000, PerkinElmer, Waltham, MA, USA) to determine the fiber content within the composite material. An appropriate amount of granular sample was placed into the instrument. The sample particles were analyzed at a heating rate of 10 °C/min within the temperature range of 30 °C to 800 °C under a nitrogen atmosphere. The residual mass percentage was used to quantify the carbon fiber content [21].

The fracture surfaces were observed using a scanning electron microscope (Sigma 300, ZEISS, Oberkochen, Germany) at an acceleration voltage of 5 kV. Samples for SEM observation were prepared by sputtering a thin layer of gold onto the surface of the obtained fracture specimens to enhance SEM visualization.

The density of the sample was measured using a high-resolution digital balance (BAS224S-CW, Sartorius, Goettingen, Germany). Based on the measured density, its porosity was calculated using the theoretical-measured density deviation method:

(1) $$P=(1-\frac{\rho }{\rho_{\mathrm{th}}})\times 100\%$$

Here, *P* denotes porosity, *ρ*_th_ represents theoretical density, and *ρ* indicates the density of the printed sample.

A universal testing machine (UTM4204, Shenzhen Suns Technology Co., Ltd., Shenzhen, China) equipped with a 20 kN force sensor was used. Tensile tests were conducted at a crosshead speed of 25 mm/s according to ASTM D638 standard for plastic tensile testing, and three-point bending tests were performed at a crosshead speed of 5 mm/s in accordance with ASTM D7264 standard for flexural performance testing. Notched impact tests were performed on an impact testing machine equipped with a 5.5 J cantilever pendulum (PTM7151, Shenzhen Suns Technology Co., Ltd., Shenzhen, China) according to ASTM D256. To minimize measurement errors, at least 5 valid data points were required per specimen group to calculate the mean and standard deviation.

## 3. Results

### 3.1. Surface Chemistry and Wettability Characterization of Different CFs

**Figure 3:**
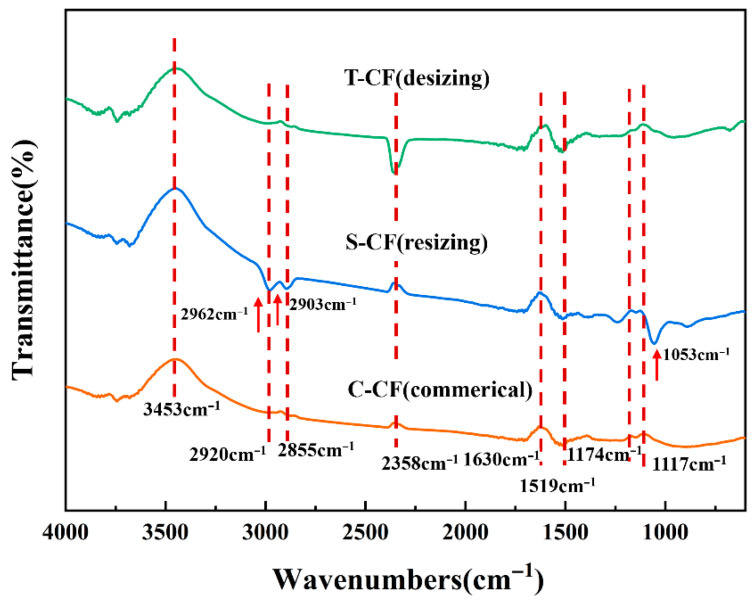
FTIR spectra of C-CF, T-CF and S-CF samples.

The surface chemical functional groups of CFs under different treatments were investigated via FT-IR, with results shown in Figure 3. CFs in all three cases exhibited peaks near ~3453 cm^−1^, attributed to the stretching vibration of O-H in intermolecular hydrogen bonds, resulting in broad peaks. The absence of significant peak fluctuations for C-CF within the range indicates that the fiber surface exhibits chemical inertness. S-CF displays two new peaks at ~2962 cm^−1^ and ~2903 cm^−1^, corresponding to the asymmetric stretching vibrations of -CH_3_ and -CH_2_ groups. These may originate from the alkyl chains in the silane coupling agent, confirming the successful sizing of KH792 onto the CF surface. T-CF exhibits an interference peak at ~2358 cm^−1^, attributed to the asymmetric stretching vibration of environmental CO_2_. Corresponding amide C=O (amide I band) and amide N-H (amide II band) [22,23] bending vibrations are found near 1630–1519 cm^−1^, along with aromatic ring C=C stretching. Multiple peaks between 1519 and 1174 cm^−1^ primarily correspond to bending vibrations of methyl (-CH_3_) and methylene (-CH_2_) groups. The peak at 1174–1117 cm^−1^ originates from C–O functional groups forming strong interactions via hydrogen bonds or covalent bonds, confirming that the pre-sizing surface oxidation treatment introduced oxygen-containing polar groups. Additionally, the new peak at ~1053 cm^−1^ corresponds to the -Si-O-Si- stretching band [24,25], further indicating that self-condensation reactions occurred between KH792 molecules [26,27]. Concurrently, intermolecular physical adsorption further immobilized the silane coupling agent onto the CF surface. All observations collectively validate the successful coating of the silane coupling agent onto the CF surface.

**Figure 4:**
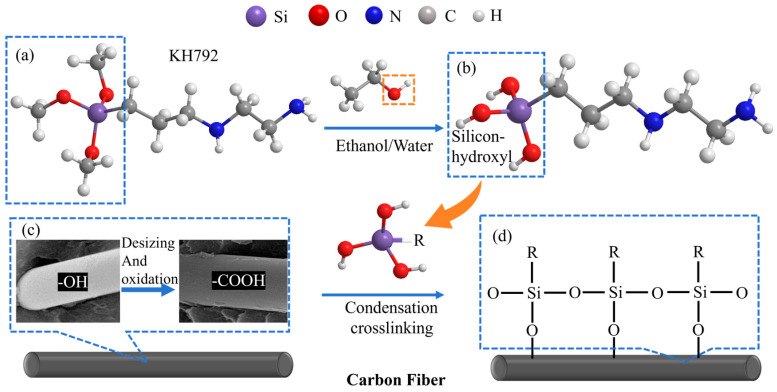
Chemical Mechanism of Carbon Fiber Treatment. (**a**) the methoxy groups (-OCH_3_), (**b**) silicon-hydroxyl, (**c**) desizing and oxidation and (**d**) Si-O-Si bonds and Si-O-C bonds.

Based on these FT-IR observations, the interfacial modification mechanism of KH-792 on the carbon fiber surface can be elucidated as follows in Figure 4. The modification treatment with KH-792 was conducted in a mixed solvent of water and ethanol. In this system, the methoxy groups (-OCH_3_) at the ends of the KH-792 molecules first undergo hydrolysis, generating highly reactive silanol groups (-SiOH) [28,29]. Ethanol, acting as a co-solvent, not only promotes the uniform dispersion of the coupling agent molecules but also effectively inhibits excessive self-condensation, ensuring a stable and thorough hydrolysis reaction.

Upon immersion of the pretreated carbon fibers into this hydrolyzed solution, the oxygen-containing functional groups on the fiber surface chemically react with the hydrolyzed silanol groups from KH-792. This reaction proceeds via two pathways: on one hand, the silanol groups undergo dehydration condensation with hydroxyl or other groups on the fiber surface, forming stable Si-O-C covalent bonds that anchor the coupling agent molecules onto the fiber surface [30,31]. On the other hand, as confirmed by the -Si-O-Si- stretching band at ~1053 cm^−1^, silanol groups from adjacent silane molecules in the solution can also condense with each other, constructing a network structure characterized by Si-O-Si bonds [25,27]. Consequently, an organic-inorganic hybrid interfacial layer, combining both chemical bonding and physical coating characteristics, is successfully built on the carbon fiber surface. The organic amino groups (-NH_2_) exposed on the outer side of this interfacial layer can interact with the nitrile groups or butadiene segments of the ABS resin matrix through hydrogen bonding or other mechanisms [25].

The wettability of untreated and sized CF was evaluated via contact angle measurement. The contact angle characterizes the equilibrium state at the liquid–solid–gas triple phase boundary, determined by the balance of interfacial tensions at this interface. Its fundamental principle is based on Young’s Equation, expressed as follows [32,33,34]:

*γ_SV_* = *γ_SL_* + *γ_LV_*•*cosθ* (2)

**Figure 5:**
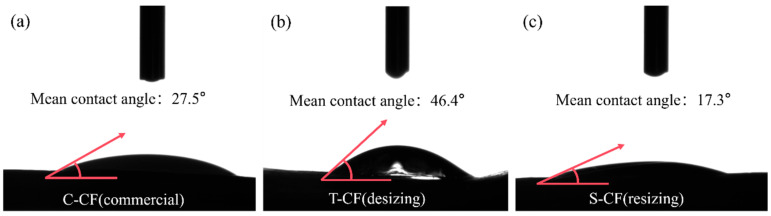
The mean contact angle of different CFs. (**a**) C-CF (commercial); (**b**) T-CF (desizing); (**c**) S-CF (resizing).

Among these, *γ_SV_*, *γ_SL_*, and *γ_LV_* represent the interfacial tensions between solid–gas, solid–liquid, and liquid–gas interfaces, respectively. Excellent wetting properties ensure good fiber–matrix interface bonding. Figure 5a–c show optical micrographs of liquid droplets on C-CF, T-CF, and S-CF substrates. The surface of C-CF presents a smooth morphology due to its original epoxy resin coating. In contrast, the desized T-CF, after immersion in acetone, exhibits distinct dispersion of dry filaments with obvious surface roughness. For the resized S-CF, its surface is re-coated with a new sizing agent layer, showing a compact and uniform smooth surface again. The results show that the average contact angle of droplets on C-CF is 27.5°, on T-CF is 46.4°, and on S-CF is 17.3°. This indicates that the contact angle of the resizing treated CF surface has significantly decreased from >40° to <20°, demonstrating improved surface wettability. This change may be attributed to the polar functional groups in the sizing agent intervening in the bonding sites on the fiber surface, thereby increasing surface energy. This effect synergizes with mechanical interlocking and chemical bonding interactions, effectively enhancing interfacial adhesion [35].

### 3.2. TGA

**Figure 6:**
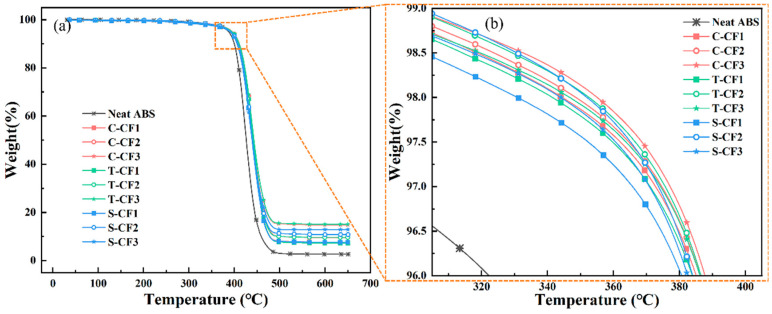
(**a**) TG curves of different content and types of CF-reinforced ABS/CF composites; (**b**) Partial magnified view of the curves.

Thermogravimetric analysis (TGA) of ABS-based composites was conducted to investigate the influence of carbon fiber on their thermal behavior. Figure 6 displays the thermogravimetric curves of carbon fiber-reinforced ABS composites with varying carbon fiber contents and different processing methods. As clearly observed in Figure 6a,b, the initial decomposition temperature of neat ABS material is approximately 343.43 °C, indicating that significant thermal decomposition begins at this temperature. As the temperature continues to rise, the neat ABS material undergoes near-complete decomposition, with the final residue mass fraction reaching only about 2.66%.

For composites incorporating CF, the onset decomposition temperature was significantly elevated, reaching up to approximately 393.59 °C for C-CF3 and 389.02 °C for S-CF3. This indicates that the addition of carbon fiber positively enhances the thermal stability of the ABS matrix, delaying the onset of thermal decomposition at higher temperatures. As the carbon fiber content increases, the decomposition temperature of the composite does not show a significant further increase. Additionally, the residual weight of the composite is markedly improved. Analysis of the residual weight reveals that the carbon fiber content for the three different ratios (CF1, CF2, CF3) are approximately 7.66%, 9.62%, and 14.79%, respectively. This indicates that carbon fiber in composite materials can resist high-temperature decomposition to a certain extent, thereby remaining after thermal decomposition and increasing the residual weight.

**Figure 7:**
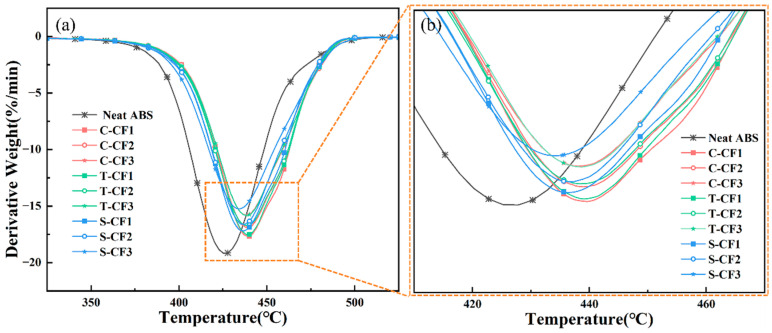
(**a**) DTG curves of different content and types of CF-reinforced CF/ABS composites; (**b**) Partial magnified view of the curves.

As shown in Figure 7, neat ABS reaches its maximum decomposition rate around 425.26 °C, marking the most intense stage of its thermal decomposition process. In contrast, for carbon fiber specimens subjected to different treatments and for CF/ABS composites reinforced with varying carbon fiber contents, the maximum weight loss rates all occur above 435 °C. This further demonstrates that the incorporation of carbon fiber significantly enhances the thermal stability of the composites, delaying the onset of maximum weight loss to higher temperatures. The TGA test results are presented in Table 2. Given that the mass fraction of neat ABS thermogravimetric residue is relatively low, this part of the residue is not included in the table.

As shown in Table 1, the residual fiber contents determined by thermogravimetric analysis are approximately 7.66% for the CF1 composite, 9.62% for the CF2 composite, and 14.79% for the CF3 composite. As shown in Table 2, all CF-reinforced composites exhibit substantially higher thermal stability than neat ABS, as evidenced by increased T_onset and T_max values. A closer examination of the DTG data reveals that, at equivalent fiber loadings, S-CF composites show slightly lower T_onset and T_max values compared to their C-CF and T-CF counterparts. This behavior of S-CF composites is attributed to the thermal decomposition of the KH-792 coupling agent itself. Silane coupling agents, including KH-792, typically decompose in the range of 350–450 °C, releasing volatile products that temporarily increase the local degradation rate and cause a slight leftward shift of the DTG peak, a phenomenon well documented by Lv et al. [36]. Additionally, Zegaoui et al. [37] reported similar phenomena in silane-modified carbon fiber composites, observing that the early-stage decomposition of the organic component coexists with improved high-temperature stability due to enhanced charring. Therefore, the KH-792 sizing agent is not detrimental to the thermal stability of the composite; on the contrary, its decomposition contributes to the formation of a more stable char layer, resulting in a net benefit for high-temperature performance.

Among carbon fiber-reinforced composites processed by different methods, C-CF/ABS and T-CF/ABS composites exhibit similar maximum weight loss rates across varying carbon fiber contents. This suggests that both processing methods have comparable effects on the interaction between carbon fibers and the ABS matrix, as well as on thermal stability. However, the maximum weight loss rate of S-CF/ABS composites was higher than that of C-CF/ABS and T-CF/ABS, suggesting that surface-treated carbon fiber may exhibit more effective interaction with the ABS matrix, thereby further enhancing the thermal stability of the composite material. Additionally, the curve of the S-CF/ABS composite exhibits a leftward shift in peak temperature, which may be attributed to the slight decomposition of the sizing agent at 435 °C. Sizing agents are typically used to enhance the surface properties and processability of carbon fibers, but they may undergo some degree of decomposition at elevated temperatures, thereby influencing the thermal decomposition curve of the composite material.

### 3.3. Mechanical Properties and Microstructure of ABS Composite Injection-Molded Specimens

**Figure 8:**
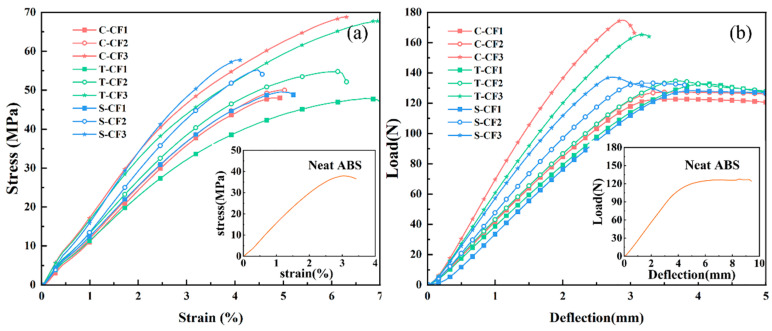
(**a**) The tensile stress–strain curves, and (**b**) the flexural load–deflection curves of the ABS-CF composites and neat ABS injection-molded specimens.

The effects of sizing agent conditions and CF content on the tensile, flexural, and impact properties of CF/ABS composites were investigated. Figure 8a shows the stress–strain curve relationships for ABS-M-CFX composite material and neat ABS material. It can be observed that neat ABS exhibits superior ductility. After adding CF, all curves exhibit brittle fracture characteristics, declining after reaching peak load, and can withstand loads far exceeding the range of neat ABS, indicating a significant increase in yield strength. Among these, ABS-S-CFX exhibited higher strength than untreated CF samples, indicating that resizing achieves both improved elongation and sufficient load-bearing capacity. Figure 8b displays the bending load–deflection curve relationships obtained from three-point bending tests on injection-molded specimens of ABS-M-CFX composites and neat ABS material. The curves transition from an initial linear increase phase to a nonlinear increase phase, followed by fracture behavior.

**Figure 9:**
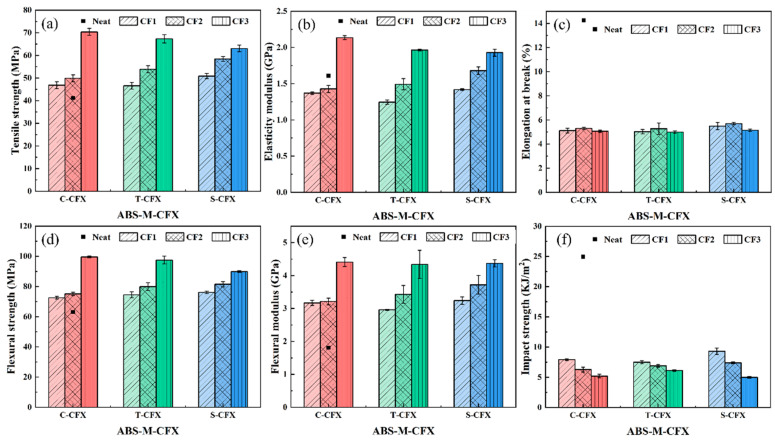
The effect of different carbon fiber mechanical properties on injection molded specimens. (**a**) Tensile strength, (**b**) elasticity modulus, (**c**) elongation at break, (**d**) flexural strength, (**e**) flexural modulus, (**f**) impact strength. Color codes represent different fiber-modified samples (red: C-CFX; green: T-CFX; blue: S-CFX).

Figure 9a,b present the tensile strength and elasticity modulus results for all tested samples. It can be observed that the tensile strength and elastic modulus of neat ABS material are the lowest, at 41.25 ± 1.57 MPa and 1.61 ± 0.02 GPa, respectively. The addition of CF enhances both strength and modulus, showing an overall upward trend compared to neat ABS. As the carbon fiber content increases, the tensile properties of all three composite materials exhibit a trend toward enhanced strength. This result indicates that the high strength and stiffness characteristics of CF exert a significantly beneficial effect on the tensile properties of the ABS-based composite material upon incorporation. The figure shows a comparison of tensile strength and elasticity modulus curves for three types of carbon fiber-reinforced ABS composites with different carbon fiber contents. The results indicate that, at a given carbon fiber content, all specially treated ABS-S-CFX samples exhibit outstanding tensile properties, with significant improvements in both tensile strength and elasticity modulus.

When the fiber content was CF2 (9.62 wt%), the tensile strength of the S-CF2 sample reached 58.40 MPa, while the tensile strengths of C-CF2 and T-CF2 were 49.90 MPa and 53.89 MPa, respectively. This indicates that at this carbon fiber content, the interfacial bonding between fibers and matrix in the S-CF2 sample is optimal, enabling efficient stress transfer and resulting in higher tensile strength. However, as the carbon fiber content continued to increase, the tensile strength of the S-CF3 sample showed a slight increase, though at a lower rate than that of the C-CF3 and T-CF3 samples. The tensile strength of C-CF3 reached 70.36 MPa, while that of T-CF3 was 67.33 MPa. In contrast, S-CF3 only achieved 63.02 MPa. This phenomenon may be attributed to changes in fiber–matrix interface bonding. As carbon fiber content increases, enhanced fiber–fiber interactions may induce stress transfer hysteresis [38,39,40], thereby affecting the tensile strength of the composite material.

This behavior can be further understood by considering the critical fiber volume fraction and percolation threshold concepts. At a fiber loading of 14.79 wt% (approximately 9.0 vol%, ρ_CF ≈ 1.8 g/cm^3^ and ρ_ABS ≈ 1.02 g/cm^3^), the fiber content approaches or exceeds the critical fiber volume fraction for fiber–fiber contact and network formation in this composite system. When the fiber volume fraction exceeds the percolation threshold (typically 5–10 vol% for high-aspect-ratio fibers), a connected fiber network forms, and the dominant stress transfer mechanism shifts from the fiber–matrix interface to fiber–fiber interactions within the network. In this regime, although the enhanced interfacial bonding in S-CF3 facilitates stress transfer at the individual fiber–matrix interface, the intensified fiber–fiber interactions cause stress to concentrate within fiber agglomerates rather than being effectively transferred to the matrix. This paradoxically leads to the observed lower tensile strength despite superior interfacial bonding. To validate this mechanism, quantitative image analysis of the SEM micrographs was performed using ImageJ software (Version 1.54g) For each specimen type (ABS-C-CF3, ABS-T-CF3, and ABS-S-CF3), threshold segmentation and particle analysis were conducted on representative SEM images at 500× magnification to characterize fiber agglomeration. The results reveal that ABS-S-CF3 exhibits the highest total agglomerate area fraction (approximately 21.8%), despite its average agglomerate size (1552 μm^2^) being intermediate between those of ABS-T-CF3 (1633 μm^2^) and ABS-C-CF3 (1229 μm^2^).

The elasticity modulus is a key indicator of a material’s resistance to elastic deformation. As shown in Figure 9b, the trend in elasticity modulus variation is similar to that of tensile strength. At a CF2 content of 9.62 wt%, the S-CF2 sample exhibits the highest elasticity modulus among the three samples with the same CF content, representing increases of 1.03 times and 1.17 times compared to the commercial CF and desized CF samples, respectively. This further demonstrates that at this carbon fiber content, the S-CF2 sample exhibits the strongest fiber–matrix interface bonding, enabling it to effectively resist elastic deformation. When the carbon fiber content increased to CF3 (14.79 wt%), the elasticity modulus of the S-CF3 sample was 1.92 GPa, lower than that of the C-CF3 and T-CF3 samples.

Figure 9d,e show the variation in the flexural properties of the composite materials with increasing CF content. Both flexural strength and flexural modulus exhibit a significant upward trend. The neat ABS matrix without CF reinforcement demonstrates the lowest performance, with flexural strength and flexural modulus values of 63.14 ± 0.97 MPa and 1.1 ± 0.07 GPa, respectively. As the carbon fiber content increases, the flexural properties of the composite material progressively improve. Among the samples, the resizing treated ABS-S-CF3 exhibited the best performance, achieving a flexural strength and flexural modulus of 89.83 MPa and 4.37 GPa, respectively—representing an increase of over 40% compared to the unreinforced ABS. This enhancement effect indicates that increased CF content expands the contact area between fibers and the matrix. Simultaneously, after removing the original epoxy resin layer and undergoing oxidation treatment, the fibers are resized, effectively opening the fiber bundles. This not only strengthens the interfacial bonding between fibers and the ABS matrix but also fully leverages the chemical bonding advantages gained from the resizing process. Additionally, the newly selected sizing agent exhibits polarity closer to the ABS matrix, enabling the formation of a complete siloxane structure on the CF surface [27,41]. This further optimizes interfacial adhesion, ensuring effective stress transfer to the fibers under bending loads and significantly enhancing the composite material’s flexural properties.

Figure 9c shows the elongation at break results for the specimens. The elongation at break for the neat ABS matrix was approximately 14 ± 0.19%, while that for the three CF/ABS composites decreased to between 4% and 5%. This indicates that the introduction of fibers caused premature interfacial debonding, leading to a significant reduction in material toughness. Figure 9f shows the variation in notch impact strength of the materials. The impact strength of neat ABS is 24.95 ± 0.15 kJ/m^2^. After adding CF, the impact strength of the composite material exhibits a significant decrease. At the lowest CF content, the impact strengths of the three composite materials reached their respective maximum values of 7.91 kJ/m^2^, 7.49 kJ/m^2^, and 9.31 kJ/m^2^. Among them, the S-CF sample demonstrated the best performance, indicating that resizing treatment facilitates the formation of a favorable interface between the fibers and the matrix, thereby enabling more effective absorption of impact loads. As CF content increases, the impact strength of all composite systems further decreases to 5.18 kJ/m^2^, 6.12 kJ/m^2^, and 4.99 kJ/m^2^, respectively. This degradation may stem from agglomeration caused by elevated fiber content, which induces stress concentration at fiber ends. This phenomenon promotes crack initiation and propagation, ultimately leading to diminished impact performance.

**Figure 10:**
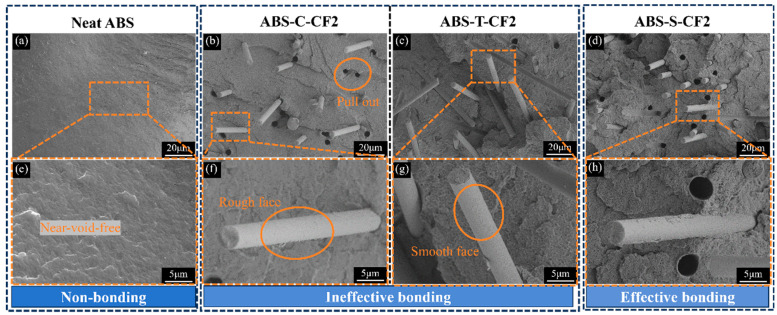
SEM images of liquid nitrogen brittle fracture surfaces of injection-molded specimens. (**a**–**d**) 500× magnification images of neat ABS and ABS-M-CF2 specimens. (**e**–**h**) 2000× magnification images of neat ABS and ABS-M-CF3 specimens.

Based on the analysis of macroscopic mechanical properties, microstructural observations of fracture morphologies in representative specimens were conducted to further elucidate the differences in interfacial behavior among various material systems. Figure 9 shows SEM images of neat ABS and three types of CF-treated ABS-M-CF2 injection-molded specimens. Figure 10a displays the brittle fracture surface of neat ABS, exhibiting numerous corrugated wrinkles on the fracture surface with no fiber pull-out marks and almost no void defects, indicating good toughness of the pure material. Figure 10b shows the brittle fracture surface of commercially available CF-reinforced ABS. It can be observed that the CF surface retains the epoxy sizing layer. Under the 2000× SEM magnification in Figure 10f, the interface between the fiber and matrix is relatively clear. The fibers are cleanly pulled out from the fracture surface without resin adhesion, indicating that the wetting–bonding between the commercial sizing agent and ABS is not ideal [42], resulting in limited load transfer efficiency. Figure 10g shows the SEM image of desizing CF-reinforced ABS at 2000× magnification. The orange dashed box highlights surface grooves on the CF, which increase surface roughness and enhance mechanical interlocking between CF and the matrix. This results in tensile and flexural strengths higher than those of commercial CF/ABS composites, as demonstrated in Figure 9a,d.

**Figure 11:**
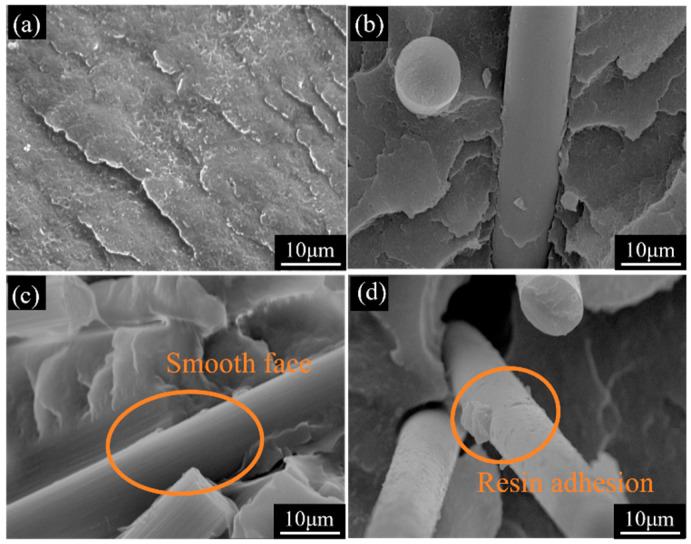
SEM images of liquid nitrogen brittle fracture surfaces of injection-molded specimens. (**a**–**d**) 5000× magnification images of neat ABS and ABS-M-CF2 specimens.

After high-temperature desizing and oxidation treatment, CF was introduced with reactive functional groups and subsequently sized using amino-functionalized KH792. Not only did the sizing agent penetrate the surface grooves of the filled CF, but the resulting amidation reaction further enhanced chemical bonding interactions, forming a composite interface that combines mechanical interlocking with chemical bonding. As shown in Figure 10d, at 2000× magnification, the surface of the CF pulled out in Figure 10h exhibits an adhered ABS resin matrix, demonstrating that the resized CFs can be successfully encapsulated by the matrix. This phenomenon indicates that the interfacial bonding performance between the fibers and the matrix has been effectively improved, which is consistent with the conclusion of enhanced mechanical properties drawn from Figure 7 and further verified by Figure 11d. By comparing Figure 10g,h, as well as Figure 11c,d, it can be observed that the ABS resin matrix provides superior encapsulation of the sizing-coated CFs. Although the unsized CFs have higher surface roughness, they rely solely on mechanical interlocking for bonding with the matrix during the pull-out process; incomplete resin encapsulation leads to stress concentration and ultimately results in premature failure of the specimens. The sizing agent exhibits polarity matching with the ABS matrix, which enhances their chemical bonding capability. Consequently, compared with desized CFs, the sizing-coated CFs demonstrate better interfacial integrity with the matrix. In summary, at a given CF content, the resized composite materials exhibit higher strength and toughness.

**Figure 12:**
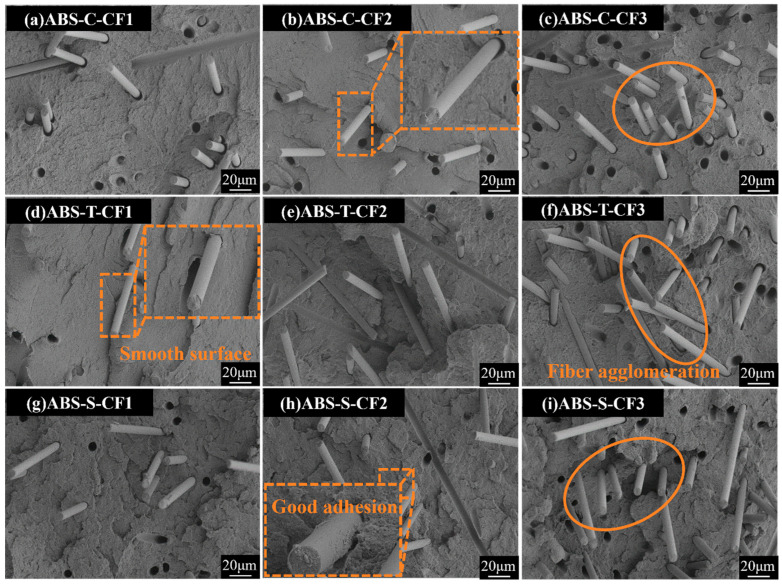
SEM images at 500× magnification of injection-molded specimens with different CF content.

Figure 12 shows SEM images of three specimens with progressively increasing carbon fiber content (CF). At CF1, the fiber spacing widens, with minimal fiber dispersion and agglomeration. The matrix forms a continuous phase, and fiber pull-out voids are scarce. At CF2, fiber density increases, revealing localized agglomerations and minor debonding voids. However, effective stress transfer between fibers remains achievable, and mechanical properties are in an ascending phase. At a CF_3_ content of CF_3_, an increase in CF debonding and pull-out cavities was observed, accompanied by more pronounced fiber agglomeration and thinning of the matrix layer. At this point, load transfer became suboptimal, and stress concentration intensified [43]. Notably, the interfacial adhesion of the resized CF system diagram significantly outperformed the previous two. Under the CF2 content diagram, the fiber surface is coated with a resin layer, indicating that the synergistic effect of chemical bonding and mechanical interlocking effectively suppresses interfacial slippage [34]. Evidently, the re-impregnated CF and ABS matrix exhibits superior interfacial bonding capability. However, when fiber content becomes excessively high, the surplus fibers agglomerate and cannot be fully impregnated by the matrix, inevitably leading to interfacial defects. Therefore, while resizing can maximize interfacial strength, agglomeration issues at high CF content must still be avoided through CF content control and process optimization.

Injection molding test results indicate that the addition of carbon fiber significantly enhances the material’s tensile and flexural properties, but reduces its toughness and impact strength. Among these, the resized sample (ABS-S-CFX) exhibited optimal comprehensive properties at a fiber content of 9.62 wt%, with significantly higher tensile strength, flexural strength, and modulus compared to both commercial and desized CF-reinforced samples. This superior performance is attributed to the strong interfacial bonding formed during resizing, which enhances effective stress transfer. SEM analysis confirmed that the cross-section of resized CF exhibited a morphology where resin enveloped the fibers, with significantly enhanced interfacial bonding. However, when the CF content exceeded 14.79 wt%, all systems showed a slowdown or decline in performance gains, particularly with a marked deterioration in impact strength. SEM observations indicate that at high fiber contents, fiber agglomeration intensifies, forming stress concentration points that lead to increased defects. Therefore, optimizing fiber content and surface treatment are key to enhancing the performance of carbon fiber-reinforced ABS composites.

### 3.4. Mechanical Properties and Microstructure of ABS Composite 3D-Printed Specimens

The fused deposition modeling (FDM) 3D printing process introduces unique structural features such as voids and interlayer bonding, which may significantly impact fiber reinforcement effectiveness and final material properties. Therefore, further research will investigate the mechanical properties and microstructure of 3D-printed specimens within the same material system, comparing them with injection-molded specimens.

**Figure 13:**
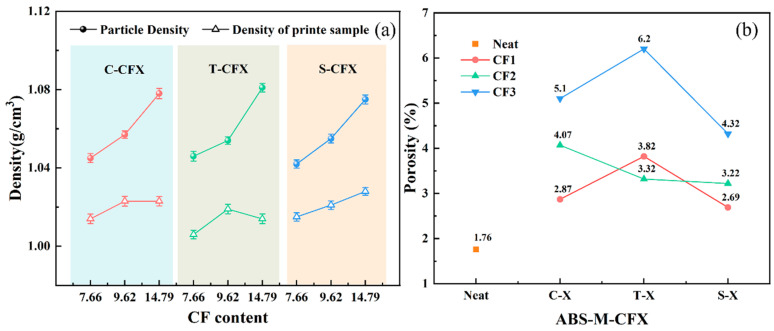
The density and porosity of the 3D-printed specimens. (**a**) Comparison between the measured sample density and particle density, (**b**) porosity of the printed specimens.

Adding fibers to pure resin materials enhances their mechanical properties, but also increases the porosity of the composite material, which further affects the overall performance of the printed product. All samples were printed under identical parameters. First, the actual density of the samples was measured based on Archimedes’ principle, with the results shown in Figure 13a. Due to the inherent layer-by-layer stacking nature of the 3D printing process, unavoidable porosity defects exist within printed samples, resulting in densities consistently lower than the theoretical maximum density achievable in injection-molded parts. This finding confirms that printed samples generally exhibit lower strength than injection-molded counterparts. Figure 13b shows the calculated porosity results for the samples. Compared to neat ABS, all carbon fiber-reinforced composites exhibited increased porosity. However, at different CF contents, the printed samples of ABS-S-CFX showed significantly lower porosity than the control groups ABS-C-CFX and ABS-T-CFX, with reductions reaching up to 29.6%, 20.9%, and 30.3%, respectively. This phenomenon indicates that the resizing process has formed a stronger interfacial bond between the carbon fibers and the ABS matrix, effectively suppressing pore formation.

To rigorously distinguish the contributions of interfacial bonding from those of porosity reduction to the mechanical properties, we normalized the tensile and flexural strengths by the measured density of each specimen (Table 3 and Table 4). After normalization, the specific tensile strength of the S-CF2 specimens was 48.35 MPa·cm^3^/g, which remained 6.1% higher than that of the C-CF2 specimens (45.59 MPa·cm^3^/g). More notably, within the CF3 series, the normalized flexural strength of the S-CF3 specimens (113.92 MPa·cm^3^/g) still exceeded that of the C-CF3 specimens (69.58 MPa·cm^3^/g) by 63.7%.

**Figure 14:**
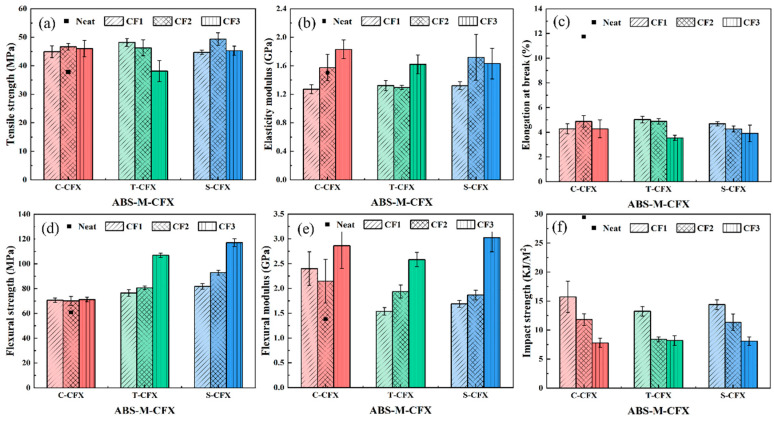
The impact of different carbon fiber mechanical properties on printed specimens. (**a**) Tensile strength, (**b**) elasticity modulus, (**c**) elongation at break, (**d**) flexural strength, (**e**) flexural modulus, (**f**) impact strength. Color codes represent different fiber-modified samples (red: C-CFX; green: T-CFX; blue: S-CFX).

Figure 14 shows the comprehensive mechanical properties of the 3D-printed samples. Compared to injection-molded samples, both tensile strength and flexural modulus exhibit certain discrepancies, primarily attributed to the porosity and interlayer bonding strength generated during the 3D printing process. Figure 14a,b present the tensile strength and elasticity modulus test results for the ABS-M-CFX composite material. As shown in the figures, the tensile strength of neat ABS is approximately 37.85 ± 1.75 MPa, with an elasticity modulus of about 1.5 ± 0.08 GPa. This makes it the group with the lowest performance among all tested samples, consistent with the characteristic of weaker intermolecular forces in neat ABS. Upon introducing carbon fiber as the reinforcing phase, both the tensile strength and elasticity modulus of the three ABS-M-CFX composites exhibited an upward trend with increasing CF content. This phenomenon primarily stems from the inherent high strength and high stiffness properties of CF itself. Acting as a load-transferring medium, CF can bear a portion of the applied external load, thereby achieving the reinforcement effect on the matrix. At the same CF content, the ABS-S-CFX system exhibits superior tensile properties: When CF content is 9.62 wt%, S-CF2 achieves the highest tensile strength and elasticity modulus among all groups with the same CF content. However, when CF content increases to CF3 (14.79 wt%), both tensile strength and elasticity modulus decrease in S-CF3. This phenomenon may be related to enhanced fiber–fiber interactions at high CF content, where fiber agglomeration readily leads to delayed stress transfer at interfaces, thereby hindering improvements in the tensile properties of the composite material.

Figure 14d,e illustrate the variation patterns of flexural strength and flexural modulus for the composite material. The flexural strength of neat ABS is approximately 60.84 ± 1.85 MPa, while its flexural modulus is about 1.38 ± 0.21 GPa. Following the introduction of carbon fiber (CF), the flexural strength and flexural modulus of the composite material significantly increased with rising CF content. Among these systems, the ABS-S-CFX system demonstrated the most pronounced reinforcement effect: at a CF content of 14.79 wt%, the flexural strength and flexural modulus of S-CF3 exceeded those of neat ABS by over 90%. Benefiting from the interface optimization effect of sizing, stress transfer to the CF reinforcing phase is effectively enhanced. Figure 14c shows the elongation-at-break results: the neat ABS exhibits an elongation at break of approximately 11.7 ± 0.31%, while all composites decrease to 4–5%, indicating that the addition of CF significantly reduces the material’s ductility. Figure 14f shows the impact strength results. The impact strength of neat ABS is 29.45 ± 0.78 kJ/m^2^, demonstrating good impact resistance. The impact strength decreases significantly after adding CF and continues to decline with increasing CF content. This is primarily due to fiber agglomeration at high CF contents, which creates stress concentration points, accelerates crack initiation and propagation, and ultimately leads to deterioration in impact performance.

**Figure 15:**
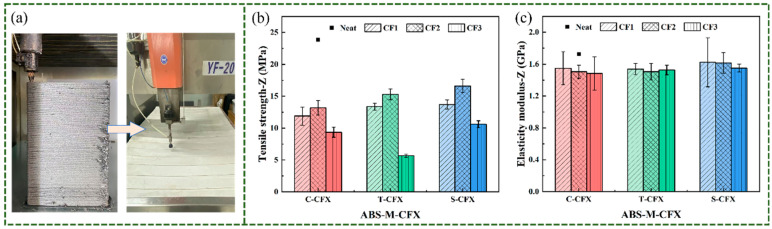
(**a**) Specimens preparation process. (**b**) Z-direction tensile strength test results. (**c**) Z-direction elasticity modulus test results. Color codes represent different fiber-modified samples (red: C-CFX; green: T-CFX; blue: S-CFX).

To evaluate the deposition fusion, we tested the tensile properties of specimens printed in the Z-axis direction. Figure 15a below shows a tensile specimen printed along the Z-axis. Due to the printing instability along the Z-axis for tensile specimens with a thickness of 3.2 mm and total length of 165 mm according to ASTM D638, their strength relies entirely on the adhesion between the upper layer of molten plastic and the lower layer of cooled, solidified plastic [44]. This bonded interface has a relatively small contact area, and the high shrinkage rate of ABS leads to weak interlayer bonding, resulting in warping and cracking. We adopted a Z-axis plate printing approach, followed by waterjet cutting to produce the specimens. Figure 15b,c show the tensile test results of the printed samples along the Z-axis. The Z-direction tensile strength of neat ABS consistently exceeded that of CF-reinforced composites. Neat ABS exhibited a tensile strength of 23.85 ± 1.05 MPa, while the highest tensile strength among CF-reinforced composites was achieved by S-CF2 at 16.61 MPa. The decrease in tensile strength following CF addition resulted from increased viscosity, which restricted melt flow and consequently led to insufficient interlayer bonding. Comparing the three CF conditions, the tensile strength of S-CF specimens was higher than that of the other two CF conditions across all CF content levels, indicating that sizing CF improved interfacial bonding strength to a certain extent. The elastic modulus of S-CF shows a slight improvement compared to the other two types, reaching approximately 1.5 GPa. However, the overall differences among the three cases are not significant. This indicates that the transverse modulus of CFs along the Z-axis is relatively low, causing the Z-axis modulus to be closer to the matrix modulus and significantly lower than the axial modulus in the X/Y directions.

It should be noted that due to the experimental challenges associated with Z-direction specimen preparation for FDM-printed composites—including printing instability, warping, and cracking caused by the high shrinkage rate of ABS—as well as the scope limitations of the current study, the results presented herein serve as a preliminary validation that surface treatment influences interlayer bonding. Among the CF-reinforced composites, the S-CF2 specimens exhibited the highest Z-direction tensile strength. A comprehensive anisotropic characterization—including systematic Z-direction testing for all CF variants and detailed analysis of fiber alignment effects on layer adhesion versus in-plane properties—will be conducted in our future work to fully elucidate the manufacturing-process-dependent behavior of these composites.

**Figure 16:**
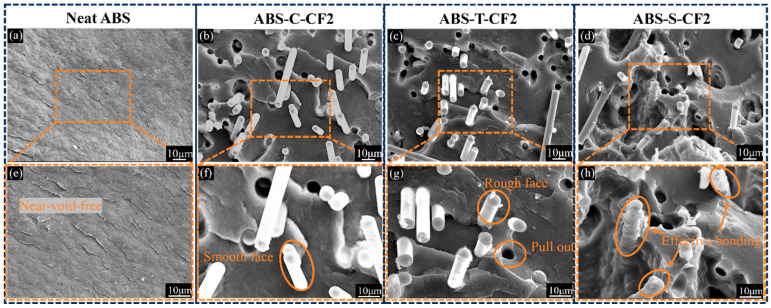
SEM images of liquid nitrogen brittle fracture surfaces of printed specimens. (**a**–**d**) 500× magnification images of neat ABS and ABS-M-CF2 specimens. (**e**–**h**) 2000× magnification images of neat ABS and ABS-M-CF3 specimens.

The aforementioned mechanical property test results, particularly the performance differences between printed and injection-molded samples, primarily stem from the effects of the printing process, resulting in fundamental differences in their microstructures. Figure 15 displays the cross-sectional SEM morphology of neat ABS and three different CF-treated ABS-M-CF2 printed specimens. Figure 16e–h show the corresponding specimens magnified at 2000×. In the high-magnification image, Figure 16f shows carbon fibers dispersed within the matrix, but the fiber surfaces appear smooth. A slight gap exists between the carbon fibers and the ABS matrix, indicating weak interfacial bonding. Figure 16g reveals rough carbon fiber surfaces with fiber pull-out phenomena, leaving distinct voids after extraction. This demonstrates insufficient interfacial bonding, causing the carbon fibers to depress the matrix and separate under external forces. Figure 16h clearly shows a uniform, dense resin matrix coating the carbon fiber surface, further confirming strong interfacial adhesion between the fibers and matrix. This enhances the load transfer capacity and macroscopic mechanical properties of the composite material.

**Figure 17:**
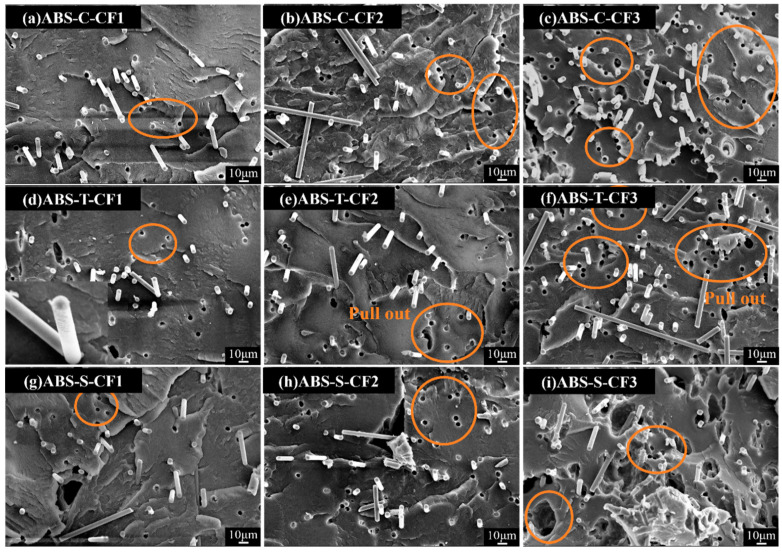
SEM images at 500× magnification of printed specimens with different CF content. The orange circled area indicates fiber pull-out.

Figure 17 shows the cross-sectional SEM morphology of the composite materials at different carbon fiber contents. For the CF1 fiber content, all three systems exhibit good fiber dispersion with relatively dense and uniform structures. When the fiber content increased to CF2, distinct fiber–matrix interface gaps were visible in the ABS-C-CF sample. The ABS-T-CF sample exhibited typical fiber pullout, indicating insufficient interfacial bonding. In contrast, the ABS-S-CF sample maintained tight interfacial bonding, with the fiber surface effectively encapsulated by the matrix. When the CF content was further increased to CF3, excessive fiber loading caused severe fiber agglomeration and large-sized voids in both the untreated and desized systems, resulting in a significant decline in structural uniformity. In contrast, although the resized system was affected by the high carbon fiber content, its interfacial bonding integrity remained superior to the other two systems.

## 4. Conclusions

This study systematically investigates the influence of carbon fiber surface conditions on the performance of ABS composites reinforced with carbon fibers. Commercial carbon fibers were subjected to desizing, oxidation, and silane coupling agent treatment to prepare three surface states: untreated, desized, and resized. ABS-C-CFX, ABS-T-CFX, and ABS-S-CFX composite specimens were fabricated using injection molding and 3D printing processes. Experiments revealed through contact angle testing that resizing treatment significantly improves the wettability of carbon fiber surfaces and enhances interfacial compatibility with the ABS matrix. SEM observations further confirmed that resized carbon fibers formed a coated bonded interface with the matrix, whereas the other two types of carbon fibers exhibited distinct interfacial defects, and this interfacial advantage was more prominent in injection-molded specimens. Mechanical property test results indicate that the resizing system exhibits optimal comprehensive performance at a fiber content of CF2 (9.62 wt%): injection-molded specimens achieved tensile strength and flexural strength of 58.41 MPa and 81.51 MPa, respectively, representing increases of 41.6% and 29.1% compared to neat ABS. The 3D-printed specimens exhibited a similar trend, achieving tensile strength and flexural strengths of 49.37 MPa and 80.19 MPa, respectively, with overall performance lower than that of injection-molded specimens. Specifically, the tensile strength was about 18.3% lower and the flexural strength was about 1.6% lower. This difference was mainly due to the interlayer defects and pores caused by the layer-by-layer stacking of 3D printing, whereas the high-pressure filling process of injection molding could achieve tighter bonding between fibers and the matrix. However, studies have also found that fiber content requires careful control. When the fiber content reaches CF3 (14.79 wt%), fiber agglomeration and increased porosity occur, leading to intensified stress concentration at the interface. This, in turn, weakens the strength and toughness of the composite material. In 3D printing processes, this phenomenon is even more pronounced, as weak interlayer bonding and inherent porosity further diminish the mechanical properties of printed specimens. Research indicates that resizing with silane coupling agents significantly enhances the interfacial bonding between carbon fibers and the ABS matrix through the synergistic effects of chemical bonding and mechanical interlocking, thereby improving the overall performance of the composite material.

## Figures and Tables

**Table 1:** Actual content of CF in 9 kinds of ABS/CF composites.

Model	CF Content	Model	CF Content	Model	CF Content
ABS-C-CF1	7.66%	ABS-T-CF1	7.66%	ABS-S-CF1	7.66%
ABS-C-CF2	9.62%	ABS-T-CF2	9.62%	ABS-S-CF2	9.62%
ABS-C-CF3	14.79%	ABS-T-CF3	14.79%	ABS-S-CF3	14.79%

**Table 2:** TGA summary of neat ABS and CF/ABS composites with different surface treatments and fiber loadings.

Samples	T_onset (°C)	T_max (°C)	dW/dt_max
Neat ABS	343.43	425.26	−17.83
ABS-C-CF1	392.86	438.87	−17.64
ABS-C-CF2	393.48	438.71	−16.86
ABS-C-CF3	393.59	438.15	−15.72
ABS-T-CF1	391.87	438.39	−17.51
ABS-T-CF2	393.16	438.21	−16.71
ABS-T-CF3	392.45	438.47	−15.82
ABS-S-CF1	389.18	436.93	−17.17
ABS-S-CF2	391.32	436.58	−16.61
ABS-S-CF3	389.08	436.03	−15.14

**Table 3:** Density-normalized tensile strength results.

Specimens	Tensile Strength (MPa)	Density (g/cm^3^)	Density-Normalized Tensile Strength (MPa·cm^3^/g)
Neat	37.85	1.002	37.78
ABS-C-CF1	44.94	1.014	44.32
ABS-C-CF2	46.64	1.023	45.59
ABS-C-CF3	46.05	1.023	45.01
ABS-T-CF1	48.19	1.006	47.90
ABS-T-CF2	46.28	1.019	45.42
ABS-T-CF3	38.14	1.014	37.61
ABS-S-CF1	44.71	1.015	44.05
ABS-S-CF2	49.37	1.021	48.35
ABS-S-CF3	45.28	1.028	44.05

**Table 4:** Density-normalized flexural strength results.

Specimens	Flexural Strength (MPa)	Density (g/cm^3^)	Density-Normalized Flexural Strength (MPa·cm^3^/g)
Neat	60.84	1.002	60.72
ABS-C-CF1	70.66	1.014	69.68
ABS-C-CF2	70.11	1.023	68.53
ABS-C-CF3	71.18	1.023	69.58
ABS-T-CF1	76.43	1.006	75.97
ABS-T-CF2	80.58	1.019	79.08
ABS-T-CF3	106.96	1.014	105.48
ABS-S-CF1	81.79	1.015	80.58
ABS-S-CF2	92.98	1.021	91.06
ABS-S-CF3	117.11	1.028	113.92

## Data Availability

The data presented in this study are available on request from the corresponding author.
